# Influences of laparoscopic-assisted gastrectomy and open gastrectomy on serum interleukin-6 levels in patients with gastric cancer among Asian populations: a systematic review

**DOI:** 10.1186/s12876-015-0276-4

**Published:** 2015-04-28

**Authors:** Zhen-Bo Shu, Hai-Ping Cao, Yong-Chao Li, Li-Bo Sun

**Affiliations:** 1 Department of Gastrointestinal Surgery, China-Japan Union Hospital, Jilin University, Xiantai Main Street No.126, Changchun, 130033 China; 2 Department of Nephrology, China-Japan Union Hospital, Jilin University, Changchun, 130033 China

**Keywords:** Laparoscopic-assisted gastrectomy, Open gastrectomy, Gastric cancer, Interleukin-6, Serum levels, Inflammatory reaction, Clinical cohort studies, Meta-analysis

## Abstract

**Background:**

To compare the effects of laparoscopic-assisted gastrectomy (LAG) and open gastrectomy (OG) on serum interleukin-6 (IL-6) levels in gastric cancer (GC) patients from Asia.

**Methods:**

The following scientific literature databases were searched for relevant clinical studies: PubMed, EBSCO, Ovid, Wiley, Web of Science, Cochrane library, EMBASE, WANFANG and VIP databases. The studies retrieved from database searches were screened based on stringent inclusion and exclusion criteria to select high quality cohort studies for the present meta-analysis. The data extracted from final selected studies were analyzed using STATA 12.0 software.

**Results:**

A total of 54 studies were initially retrieved from database searches, and 11 clinical cohort studies were eventually enrolled in this meta-analysis. The 11 selected studies contained a combined total of 767 GC patients (427 patients in LAG group and 340 patients in OG group). Meta-analysis results demonstrated that postoperative serum IL-6 levels in GC patients in LAG group was significantly lower than the OG group (SMD = −2.16, 95% CI = −3.19 ~ −1.14, *P* < 0.001). The difference in serum IL-6 levels between the preoperative and postoperative GC patients was significantly lower in the LAG group compared to the difference found in the OG group (SMD = −3.44, 95% CI = −4.87 ~ −2.01, *P* < 0.001). Subgroup analysis based on country showed that, in both Chinese and Japanese GC patients, the postoperative increase in serum IL-6 levels in LAG group were significantly lower than the increase observed in the OG group (all *P* < 0.05). In Korean GC patients, the postoperative increase in serum IL-6 levels was not significantly different between the LAG group and OG group (all *P* > 0.05).

**Conclusion:**

Our results provide strong evidence that LAG is associated with significantly lower serum IL-6 levels, compared to OG. Thus, LAG carries markedly lower risk of adverse inflammatory reactions in GC patients among Asian population.

## Background

Gastric cancer (GC) is a cancer originating from the tissue lining of the stomach. Despite a worldwide decrease in the incidence of GC in the past 40 years, GC remains the fourth most common cancer and the second major cause of cancer-related death globally[1,2]. GC is uncommon in the United States and Europe, but is much more common in China and Japan, and other Asian countries. Apart from Asia, GC also has a higher incidence in South America [3].Surgical resection by gastrectomy is the only option for GC treatment that is sufficient to cure early-stage gastric cancer patients, and significantly enhance patient survival in more advanced GC, when combined with radiation therapy and chemotherapy. Among the options for gastrectomy, open gastrectomy (OG) has traditionally been widely used [4]. Laparoscopic-assisted gastrectomy (LAG), involving the use of laparoscopic surgery and its related equipment, was developed as a minimally invasive approach and has also been in use since its first description in 1999 for treatment of GC [5,6]. However, LAG has limited field of vision compared to OG and the choice between OG and LAG is much debated in literature, with no firm conclusions. Notably, a recent study found that interleukin-6 (IL-6) is a major factor in peritoneal immune response after surgery in GC patients, and surgical options for GC will need to be re-evaluated in light of the potentially adverse influence of inflammatory responses on therapy safety and effectiveness [7].

IL-6 is a 26 kDa protein initially described as a B cell activating factor produced by T cells, and is mapped to 7p15-p21 chromosome [8]. As a dual-property cytokine with pro-inflammatory and anti-inflammatory roles, IL-6 is prominently involved in inflammatory processes during host immune defense response and stimulates growth and proliferation of a variety of immune cell types, and IL-6 is regarded as a key regulator in human immune regulation and inflammatory reaction [9]. Inflammatory reaction is activated by the surgical procedures, and there is an association between the extent of the surgical trauma and the inflammatory response [10]. IL-6 serum levels are positively associated with the severity and the extent of postoperative inflammation, and IL-6 is regarded as a reliable indicator of inflammatory reaction for comparing the efficacies of OG and LAG for treatment of GC [7]. LAG is associated with lower serum IL-6 levels compared to OG [11] and the surgery-induced increase in serum IL-6 levels are significantly lower for LAG, compared to the levels induced following OG [12,13]. Apart from the lower serum IL-6 levels, LAG offers the advantages of less blood loss, reduced postoperative complications, shorter hospital stays and accelerated recovery [14]. Current studies have shown that LAG involves less surgical trauma compared with OG, and serum IL-6 levels is lower in the laparoscopic operation [15,16]. However, other studies show contrasting results [17,18]. To address this issue, we conducted the present meta-analysis to compare the effect of LAG and OG on serum IL-6 levels in Asian GC patients.

## Methods

A systematic review of meta-analysis was conducted and the results were described according to the PRISMA statement [19].

### Literature search

To retrieve relevant literature comparing the effects of LAG and OG on serum IL-6 levels in GC patients, we comprehensively searched the following electronic databases: PubMed, EBSCO, Ovid, Wiley, Web of Science, Cochrane library, EMBASE, WANFANG and VIP databases (last updated search in December 2014), without language restrictions. Keywords used for electronic databases search were: stomach neoplasms, Interleukin-6, IL-6, laparoscope gastrectomy and open gastrectomy in combination with the Boolean operators AND, OR and NOT. We also manually searched related bibliographies for studies that were missed in the initial electronic search.

### Inclusion and exclusion criteria

In this meta-analysis, studies were selected if they met the following inclusion criteria: (1) study design: clinical cohort trial; (2) study issue: the effects of LAG and OG on serum IL-6 levels in GC patients; (3) study subject: GC patients verified by endoscopy and biopsy and patients treated with LAG or OG; (4) detection method for IL-6 levels: enzyme-linked immune sorbent assay (ELISA); (5) detection times for IL-6 levels: 24 h before operation and 24 h after operation; (6) trials provided sufficient data required for our study, such as preoperative IL-6 levels and postoperative IL-6 levels; (7) studies were either Chinese or English. In cases of overlap reports, we included only the latest results. Studies were excluded if they failed to meet the inclusion criteria. Study with patients who had a history of previous treatment of GC, chemotherapy or radiation was also excluded.

### Data extraction and quality assessment

All data from eligible trials were extracted by two investigators independently using a standard form, and the following information was collected: first author, publication year, country, ethnicity, language, disease, detection method, age, gender and sample number. Critical appraisal skill program (CASP) score criteria of Oxford Center for Evidence-based Medicine was employed for methodological assessment of quality of the included cohort studies [20]. Each included study was scored on 12 aspects: (1) whether the study address a clearly focused issue (CASP01); (2) whether the cohort were chosen in an acceptable way (CASP02); (3) whether the exposure precisely measured to reduce bias (CASP03); (4) whether the outcome precisely measured to reduce bias (CASP04); (5) whether the authors identified all significant confounding factors; whether they considered confounding factors in the design or analysis (CASP05); (6) whether the follow up of subjects was complete; whether the follow up of subjects was long enough (CASP06); (7) whether the result of this study in complete (CASP07); (8) whether the result was accurate (CASP08); (9) whether the result of the study in believable (CASP09); (10) whether the result could be applied to local population (CASP10); (11) whether the result fit with other available evidence (CASP11); (12) whether this study provided implication for practice (CASP12). The quality evaluation of included studies was performed by two investigators. Any disagreement in study selection or quality assessment was resolved by further discussion.

### Statistical analysis

Statistical analyses were carried out with the STATA statistical software (Version 12.0, Stata Corporation, College Station, TX, USA). To compare the effects of LAG and OG on serum IL-6 levels in GC patients, standardized mean difference (SMD) with 95% confidence interval (95%CI) was analyzed. Z test was applied to evaluate the significance of overall effect size (SMDs) [21]. Heterogeneity among the studies were evaluated by Cochran’s Q-statistic (*P* < 0.05 was considered as evident heterogeneity) and *I*^*2*^ test which is the percentage of total variation across studies ranging from 0 to 100% [22,23]. A random effects model was applied if there was significant heterogeneity (*P* < 0.05 or *I*^*2*^ > 50%), otherwise a fixed effects model was employed [24]. Univariate and multivariate meta-regression analysis was utilized to identify potential sources of heterogeneity, and further confirmed by Monte Carlo method [25,26]. Additionally, we applied a sensitivity analysis to evaluate whether one single study had the weight to impact the overall estimate. Further, the existence of publication bias was detected by funnel plot and Egger’s linear regression test (*P* < 0.05 was considered significant) [22,27].

## Results

### The baseline characteristics of included studies

**Figure 1:**
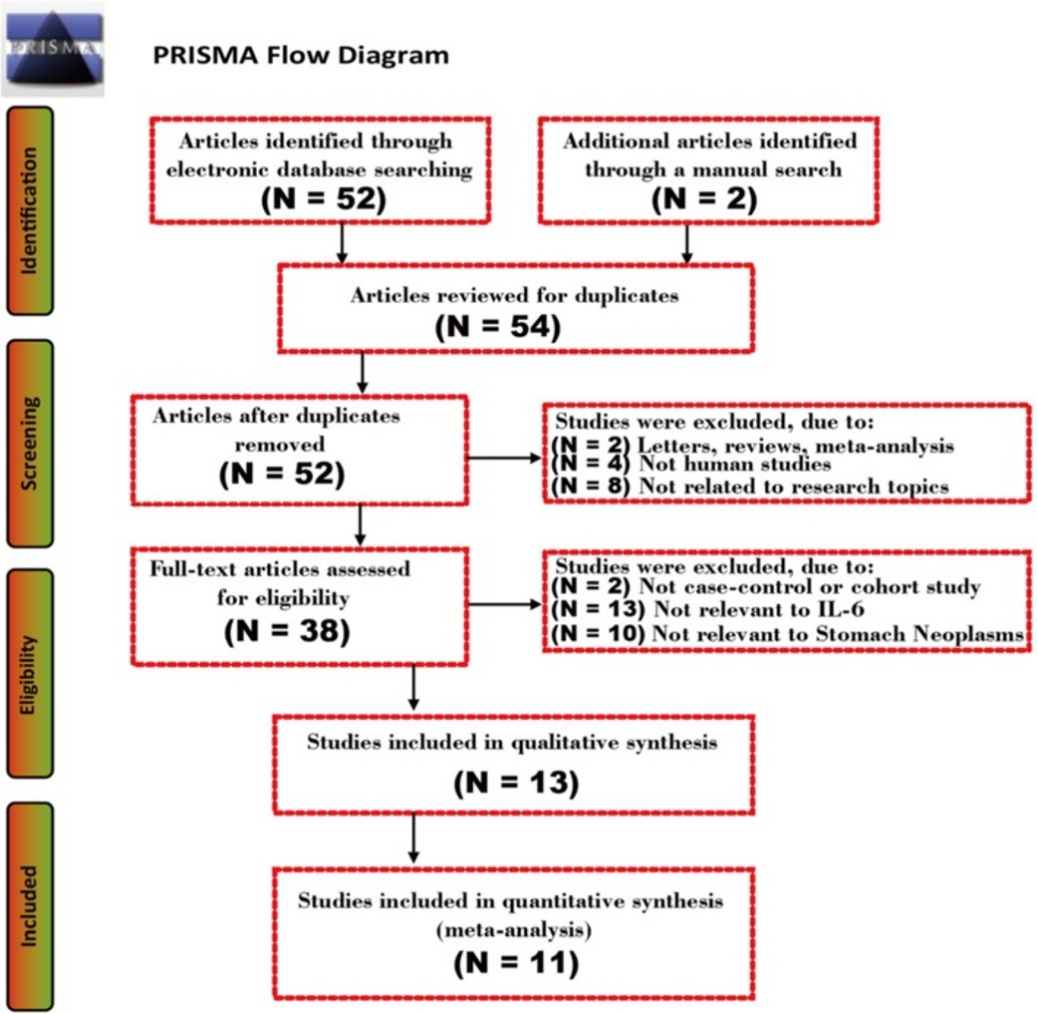
Flow chart shows the detailed study inclusion and exclusion procedures. Eleven clinical cohort studies were included in this meta-analysis.

**Figure 2:**
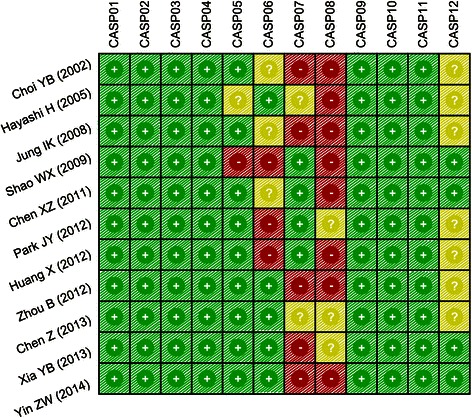
The critical appraisal skill program (CASP) score for assessing the methodological quality for the eleven enrolled clinical cohort studies.

A total of 54 studies were initially retrieved through electronic database search and manual search. Our selection criteria excluded duplicates (n = 2), letters, reviews or meta-analyses (n = 2), non-human studies (n = 4), and studies not related to research topics (n = 8). The remaining studies (n = 38) were reviewed carefully to examine the full text for data integrity. Subsequently, additional studies were excluded because they lacked sufficient data (n = 25) or did not contain high quality data (n = 2). Eventually, 11 cohort studies [5,17,18,28-35], published between 1998 and 2014, were included in this meta-analysis. The 11 clinical cohort studies contained a combined total of 767 GC patients (427 patients in LAG group and 340 patients in OG group). Within the 11 studies, 7 trials were conducted in China, 2 trials were performed in Japan and 2 trials were performed in South Korea. Figure 1 shows the flow chart of the study selection process. Demographic information and baseline characteristics of the enrolled studies and CASP score are showed in Table 1 and Figure 2, respectively.

**Table 1:** **Baseline characteristics of the enrolled 11 clinical cohort studies**

First author	Year	Country	Sample Size		Gender (M/F)		Age (years)		Method
			LAG	OG	LAG	OG	LAG	OG	
Choi YB [17]	2002	Japan	10	10	7/3	9/1	58.7 (33 ~ 80)	60.4 (25 ~ 78)	ELISA
Hayashi H [18]	2005	Japan	14	14	9/5	13/1	56 (47 ~ 70)	62 (49 ~ 75)	ELISA
Jung IK [34]	2008	Korea	10	10	4/6	9/1	54.8 ± 16.1	62.9 ± 6.6	ELISA
Shao WX [30]	2009	China	47	47	31/16	30/17	57.7 (26 ~ 77)	57.0 (28 ~ 76)	ELISA
Chen XZ [5]	2011	China	15	15	12/3	12/3	52.3 ± 12.2	54.3 ± 12.3	ELISA
Park JY [35]	2012	Korea	120	30	65/55	18/12	55 (45 ~ 64)	50 (45 ~ 54)	ELISA
Huang X [29]	2012	China	30	30	21/9	23/7	56	58	ELISA
Zhou B [33]	2012	China	45	45	24/21	25/20	51.8 ± 10.6	50.1 ± 11.4	ELISA
Chen Z [28]	2013	China	36	36	19/17	21/15	52.6 ± 4.2	53.2 ± 4.8	ELISA
Xia YB [31]	2013	China	55	59	33/22	39/20	54.0 ± 3.2	52.7 ± 1.8	ELISA
Yin ZW [32]	2014	China	45	44	29/16	33/11	55.9 ± 8.5	56.1 ± 7.9	ELISA

*M* male; *F* female; *ELISA* enzyme-linked immune sorbent assay; *LAG* laparoscopic-assisted gastrectomy; *OG* open gastrectomy.

### Comparison of postoperative serum IL-6 levels in LAG and OG groups

**Figure 3:**
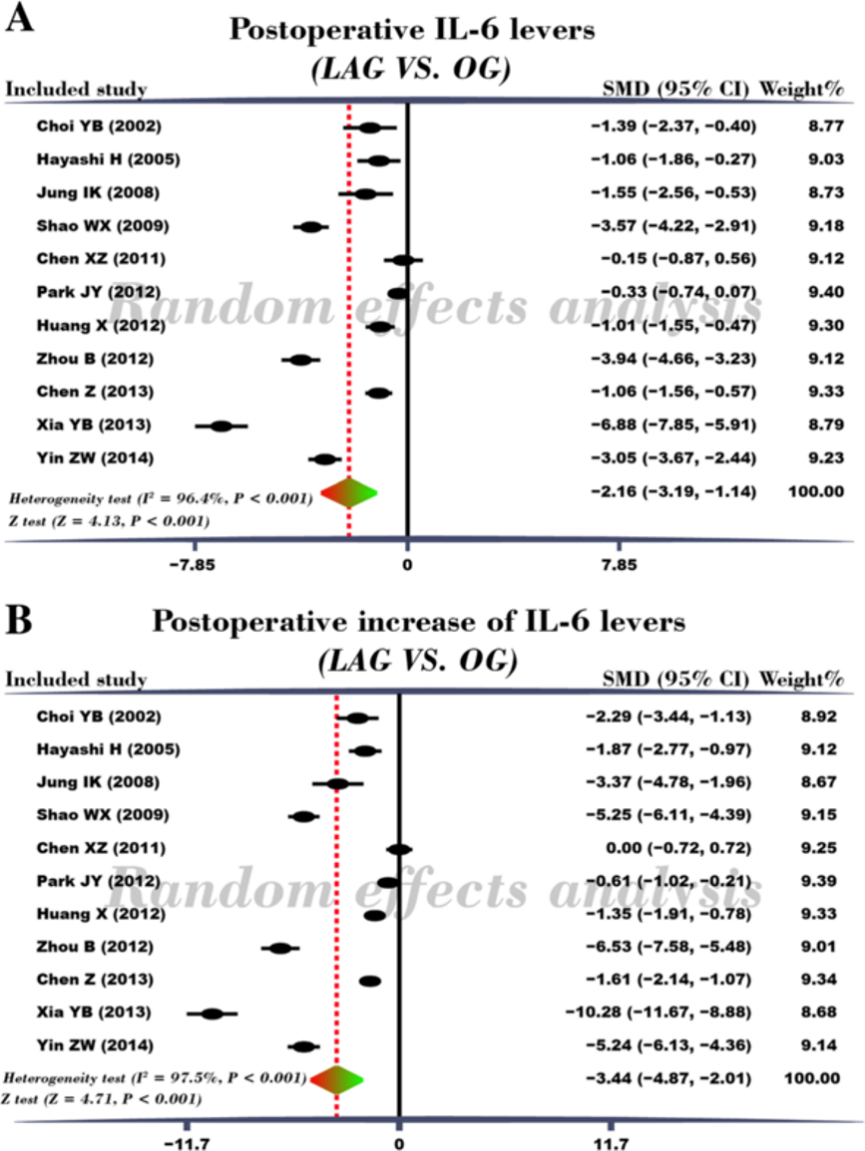
Forest plots. **(A)** Comparison of postoperative IL-6 levels (LAG group VS. OG group). **(B)** Comparison of postoperative increased IL-6 levels (LAG group VS. OG group).

**Figure 4:**
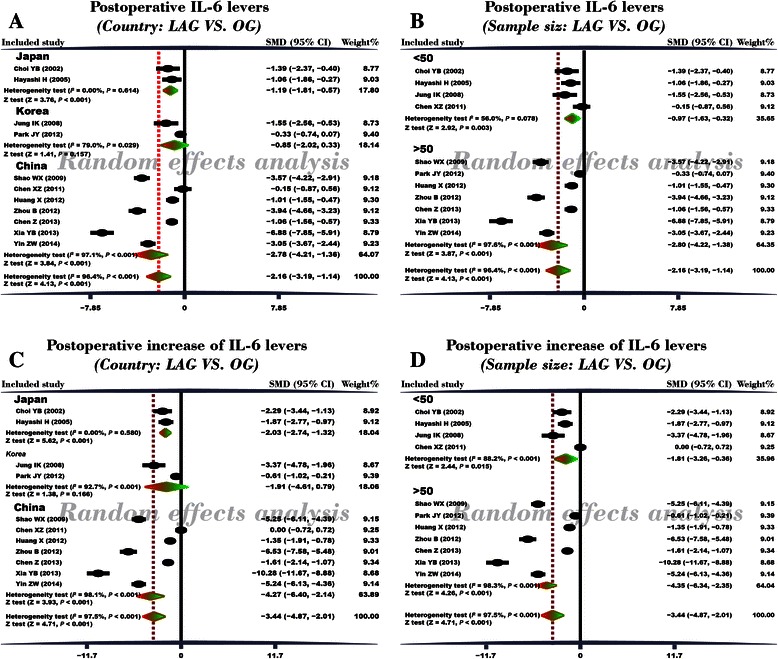
Forest plots of subgroup analyses based on country and sample size. **(A)** Subgroup analyses based on country for the comparison of postoperative IL-6 levels (LAG group VS. OG group). **(B)** Subgroup analyses based on sample size for the comparison of postoperative IL-6 levels (LAG group VS. OG group). **(C)** Subgroup analyses based on country for the comparison of postoperative increased IL-6 levels (LAG group VS. OG group). **(D)** Subgroup analyses based on sample size for the comparison of postoperative increased IL-6 levels (LAG group VS. OG group).

**Figure 5:**
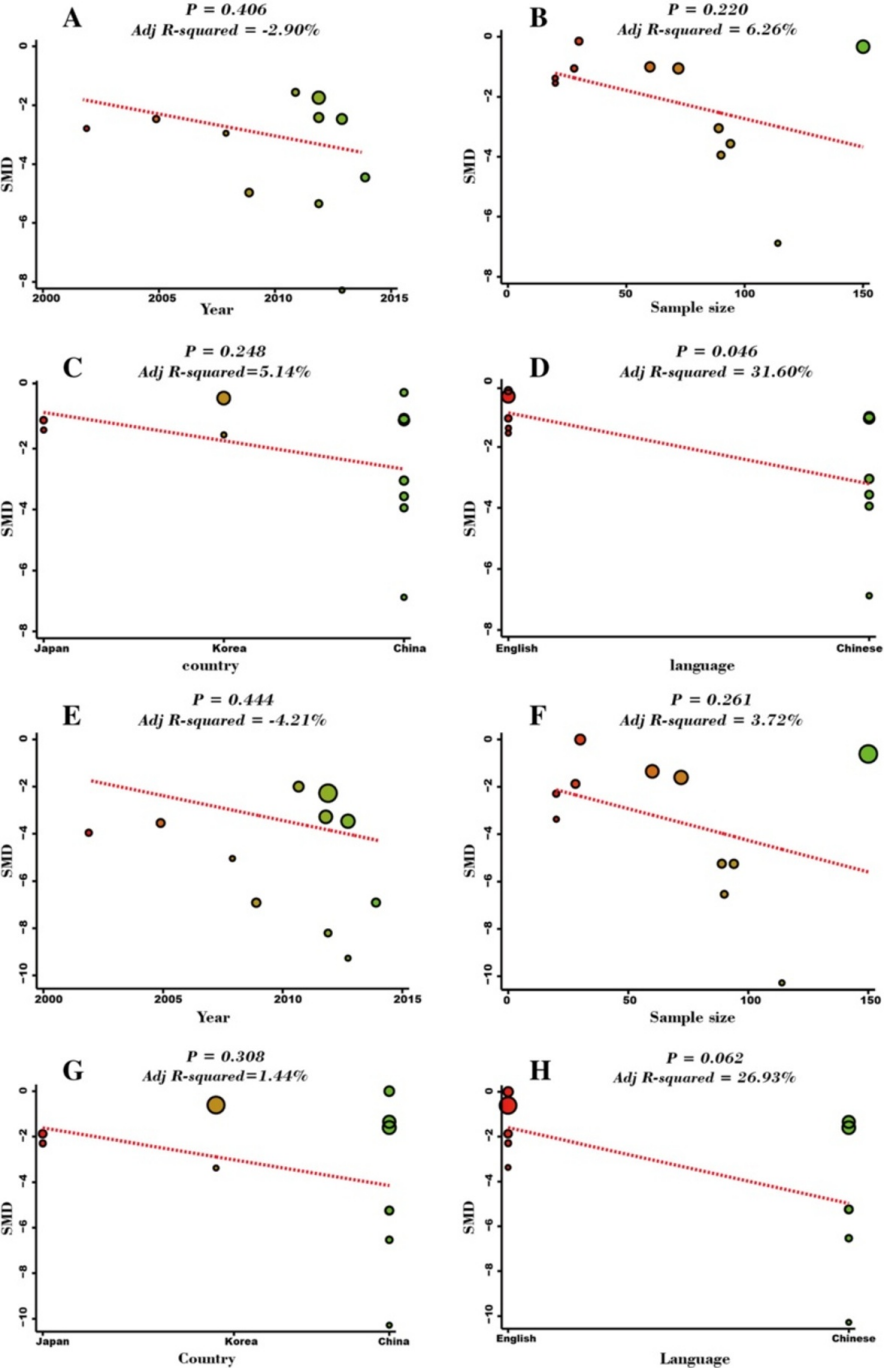
Univariate meta-regression analysis. **(A)** Univariate meta-regression analysis on Year for the comparison of postoperative serum IL-6 levels (LAG group VS. OG group). **(B)** Univariate meta-regression analysis on Sample size for the comparison of postoperative serum IL-6 levels (LAG group VS. OG group). **(C)** Univariate meta-regression analysis on Country for the comparison of postoperative serum IL-6 levels (LAG group VS. OG group). **(D)** Univariate meta-regression analysis on Language for the comparison of postoperative serum IL-6 levels (LAG group VS. OG group). **(E)** Univariate meta-regression analysis on Year for the comparison of postoperative increased serum IL-6 levels (LAG group VS. OG group). **(F)** Univariate meta-regression analysis on Sample size for the comparison of postoperative increased serum IL-6 levels (LAG group VS. OG group). **(G)** Univariate meta-regression analysis on Country for the comparison of postoperative increased serum IL-6 levels (LAG group VS. OG group). **(H)** Univariate meta-regression analysis on Language for the comparison of postoperative increased serum IL-6 levels (LAG group VS. OG group).

A random effects model was applied for existence of heterogeneity among the included studies (*I*^*2*^ = 96.4%, *P* < 0.001). The result of this meta-analysis demonstrated that postoperative serum IL-6 level of GC patients in LAG group was significantly lower than in the OG group (SMD = −2.16, 95% CI = −3.19 ~ −1.14, *P* < 0.001) (Figure 3A). Subgroup analysis based on country showed that, in Chinese and Japanese GC patients, postoperative serum IL-6 levels in LAG group were significantly lower than OG group (Japanese: SMD = −1.19, 95% CI = −1.81 ~ −0.57, *P* < 0.001; Chinese: SMD = −2.78, 95% CI = −4.21 −1.36, *P* < 0.001). In Korean GC patients, however, postoperative serum IL-6 levels in LAG group and OG group showed no statistical differences (SMD = −0.85, 95% CI = −2.02 0.33, *P* = 0.157). Further subgroup analysis based on sample size revealed that, whether the sample size was less than 50 or more than 50, the postoperative serum IL-6 levels in LAG group were significantly lower than the IL-6 levels in the OG group (sample size < 50: SMD = −0.97, 95% CI = −1.63 ~ −0.32, *P* = 0.003; sample size > 50: SMD = −2.80, 95% CI = −4.22 ~ −1.38, *P* < 0.001) (Figure 4A-B). Univariate meta-regression analysis revealed that publication year, sample size, country and language were not the potential sources of heterogeneity (all *P* > 0.05). Language is a possible source of heterogeneity, while publication year, sample size and country were not the potential sources of heterogeneity. (Figure 5A-D). Multivariate meta-regression analysis further confirmed that publication year, sample size, country and language were not the potential sources of heterogeneity (Table 2).

**Table 2:** **Meta-regression analyses for the potential source of heterogeneity**

Heterogeneity factors	Coefficient	SE	t	P(Adjusted)	95% CI	
					LL	UL
Year	0.293	0.404	0.73	0.832	−0.695	1.281
Sample Size	−0.018	0.020	−0.91	0.717	−0.104	0.031
Country	−0.500	1.902	−0.26	0.998	−7.725	4.155
Language	−2.399	1.856	−1.29	0.503	−11.084	2.141

*SE* Standard Error; *LL* Lower Limit; *UL* Upper Limit.

### Comparison of the differences between preoperative and postoperative serum IL-6 levels in LAG and OG groups

After Cochran’s Q-statistic and *I*^*2*^ tests, a random effects model was used due to the existence of heterogeneity among the included studies (*I*^*2*^ = 97.5%, *P* < 0.001). The result of this meta-analysis revealed that in LAG group, the difference between preoperative and postoperative serum IL-6 level in GC patients was lower than the difference seen in the OG group (SMD = −3.44, 95% CI = −4.87 ~ −2.01, *P* < 0.001) (Figure 3B). Subgroup analysis based on country showed that, in Chinese and Japanese GC patients, the increase in postoperative serum IL-6 levels in LAG group were significantly lower than the postoperative increases found in the OG group (Japanese: SMD = −2.03, 95% CI = −2.74 ~ −1.32, *P* < 0.001; Chinese: SMD = −4.27, 95% CI = −6.40 ~ −2.14, *P* < 0.001). In Korean GC patients, postoperative increased serum IL-6 levels in LAG group and OG group were not different (SMD = −1.91, 95% CI = −4.61 ~ 0.79, *P* = 0.166). Additional subgroup analysis based on sample size revealed that, whether studies contained sample size < 50 or > 50, the increases in postoperative serum IL-6 levels in LAG group were lower than the post-operative increases found in the OG group (sample size < 50: SMD = −1.81, 95% CI = −3.26 ~ −0.36, *P* = 0.015; sample size > 50: SMD = −4.35, 95% CI = −6.34 ~ −2.35, *P* < 0.001) (Figure 4C-D). Univariate meta-regression analysis revealed that publication year, sample size, country and language were not the potential sources of heterogeneity (all *P* > 0.05) (Figure 5E-H). Multivariate meta-regression analysis further confirmed that publication year, sample size, country and language were not the potential sources of heterogeneity (Table 3).

**Table 3:** **Meta-regression analyses of potential source of heterogeneity**

Heterogeneity factors	Coefficient	SE	t	P (Adjusted)	95% CI	
					LL	UL
Year	0.347	0.649	0.53	0.938	−1.242	1.935
Sample Size	−0.024	0.033	−0.72	0.853	−0.104	0.056
Country	−0.253	3.054	−0.08	1.000	−7.725	7.219
Language	−3.798	2.978	−1.28	0.530	−11.084	3.488

*SE* Standard Error; *LL* Lower Limit; *UL* Upper Limit.

### Sensitivity analysis and publication bias

**Figure 6:**
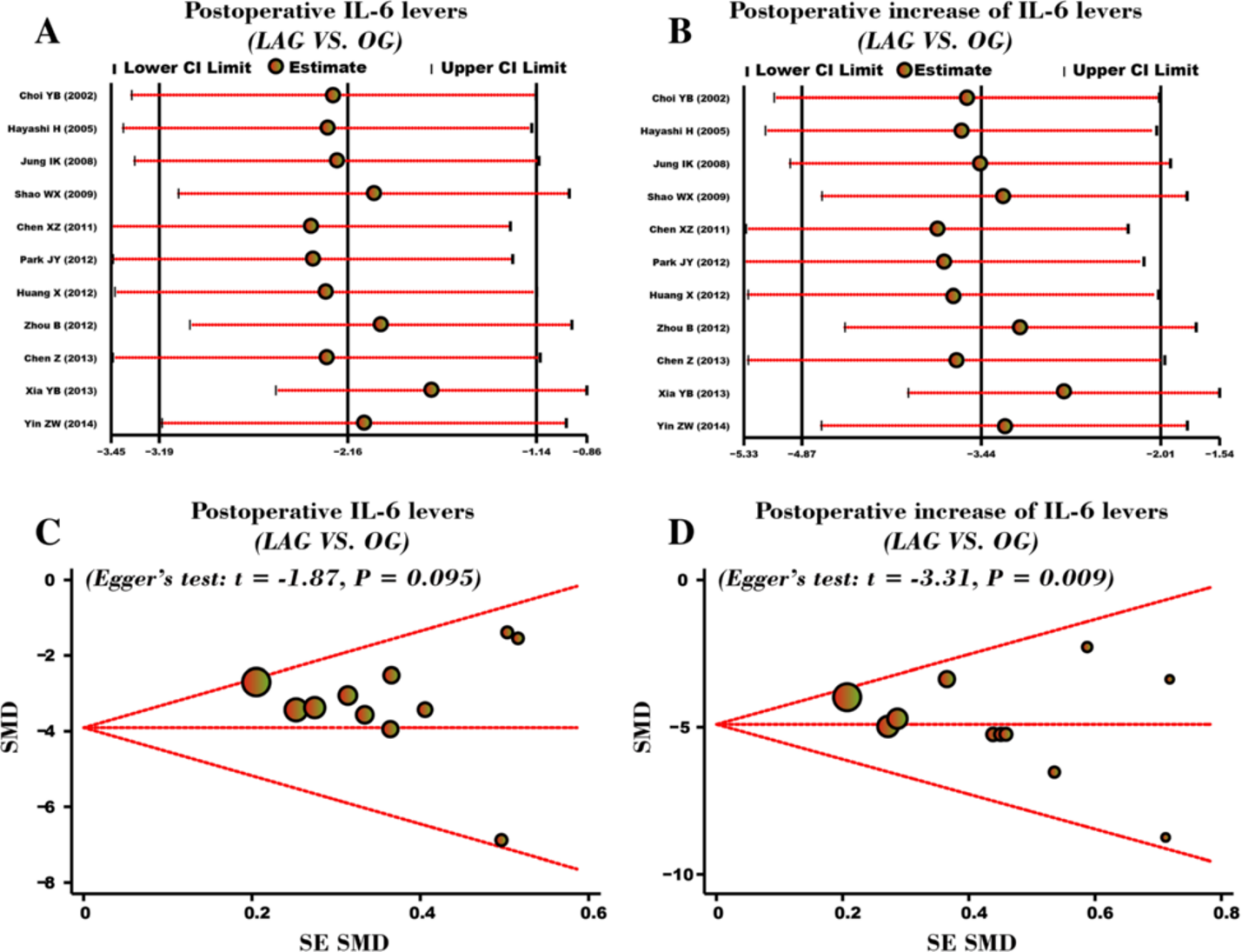
Sensitivity analysis and publication bias assessment. **(A)** Sensitivity analysis for the comparison of postoperative serum IL-6 levels (LAG group VS. OG group). **(B)** Sensitivity analysis for the comparison of postoperative increased serum IL-6 levels (LAG group VS. OG group). **(C)** Publication bias assessment for the comparison of postoperative serum IL-6 levels (LAG group VS. OG group). **(D)** Publication bias assessment for the comparison of postoperative increased serum IL-6 levels (LAG group VS. OG group).

The result of sensitivity analysis showed that any single study selected in this meta-analysis had no significant effect on the pooled SMDs (Figure 6A-B). The symmetric funnel plots for comparison of postoperative serum IL-6 levels of GC patients between LAG group and OG group suggested no publication bias in the enrolled studies (Figure 6C). The Egger linear regression analysis further confirmed the evidence of no publication bias (*P* = 0.095). The funnel plots for comparison of postoperative increased serum IL-6 levels of GC patients between LAG group and OG group presented asymmetric shape, suggesting existence of publication bias (Figure 6D). The presence of bias may have resulted from the use of pre-operative data for the analysis of postoperative increase of IL-6. The Egger linear regression analysis further confirmed the presence of publication bias (*P* = 0.009).

## Discussion

In order to examine the change in serum IL-6 level after LAG and OG treatment in GC patients, a systematic meta-analysis was performed. The main result of this meta-analysis showed that postoperative serum IL-6 level was significantly lower in LAG group compared to OG group. The increase in postoperative serum IL-6 levels of GC patients in the LAG group was also significantly lower than postoperative increases found in the OG group. IL-6 is secreted at local sites and released into the blood circulation when homeostatic perturbation occur such as endotoxemia, trauma, endotoxic lung, and acute infections [36]. IL-6 mediates inflammatory process by stimulating B cell activation, B cell differentiation, differentiation of T cell and macrophages and NK cell activation [37]. Additionally, IL-6 has a broader biological function as an adipokine and a myokine for muscle contractions, and as a neuropeptide [38]. Relevant to cancers, IL-6 activates STAT3 signaling pathways and high levels of IL-6 is associated with poor prognosis in a variety of cancers such as prostate cancer, bladder cancer, ovarian cancer, colorectal cancer and GC [39,40]. IL-6 is also an important mediator of acute-phase response, and its high serum levels in post-surgery patients correlate with the severity of surgical trauma, loss of blood, surgical duration and tissue damage [41,42]. Compared with OG, surgical stress of LAG is lower and results in reduced inflammation [43,44]. Besides, with reduced manipulation response, LAG has advantages in less blood loss and pain during the surgery, earlier wound recovery, shorter hospital stays and quicker convalescence [14,45].From the above analysis, although IL-6 serum levels increased after both OG or LAG treatments, significantly lower increases were found in LAG group, indicating that LAG treatment shows a better surgical outcome, compared to OG, with a higher safety profile and reduced inflammatory responses, which are significant advantages that influence the overall patient survival. Consistent our analysis, Adachi et al., also found that the serum IL-6 level showed a marked increase after OG or LAG treatment, but serum IL-6 level decreased more rapidly in LAG group on day 3, suggesting an additional advantage of LAG over OG [15].

The influence of other factors, such as country and sample size, on the relationship between IL-6 level and OG or LAG treatments was examined by subgroup analyses. A subgroup analysis based on country revealed that in both Chinese and Japanese GC patients, the postoperative serum IL-6 levels were significantly lower in LAG group compared to the IL-6 levels found in the postoperative OG group. In Korean GC patients, however, the postoperative serum IL-6 levels in LAG group and OG group showed no statistical differences. The possible explanation may be the influence of different life styles on relatively small number of selected studies. Thus, lower serum IL-6 level in LAG treatment, compared with OG treatment, was confirmed in our analysis, which is consistent with previous studies, suggesting that the choice of gastrectomy procedures should be carefully considered based on individual patient’s pathology and co-morbidity, to derive maximal outcomes for GC patients. In this respect, our results provide evidence that LAG is a better option for GC treatment.

There were several limitations in our present meta-analysis. First, the relatively small number of studies and the small sample size may have an influenced our statistical analysis. In the ten enrolled, only 10 patients each were reported in the study of Jung IK et al., and other studies also reported relatively small sample size. Second, the serum levels of IL-6 is associated with the degree of surgery-associated stress, nevertheless, other cytokines and hormones, such as TNF, IFN-c and catecholamines, are also elevated during surgical stress and significantly influence the overall inflammatory response, and is not just limited to IL-6. However, in this current meta-analysis, we did analyze the data related to the levels of other cytokines. Third, Figure 6C shows symmetric shape for the comparison on postoperative serum IL-6 levels of GC patients between LAG group and OG group, while Figure 6D shows asymmetric shape for the postoperative increased serum IL-6 levels of GC patients between LAG group and OG group. A contradiction exists in this results and the possible explanation may be the use of preoperative data for the analysis of postoperative increase of IL-6, resulting in the bias.

## Conclusions

Our study showed that lower serum IL-6 level is found after LAG treatment, compared with the IL-6 level found after OG treatment, suggesting that LAG is a better surgical procedure in treating GC patients, in light of the adverse consequences of IL-6 mediated inflammation in GC patients. However, further studies with more comprehensive data and larger sample-size are warranted to strengthen our results.

## Abbreviations

IL: Interleukin
LAG: Laparoscopic-assisted gastrectomy
OG: Open gastrectomy
GC: Gastric cancer
CASP: Critical appraisal skill program
SMD: Standard mean difference
